# HIOP-Reader: Automated Data Extraction for the Analysis of Manually Recorded Nycthemeral IOPs and Glaucoma Progression

**DOI:** 10.1167/tvst.11.6.22

**Published:** 2022-06-23

**Authors:** Vaia Agorastou, Julian Schön, Raoul Verma-Fuehring, Mohamad Dakroub, Jost Hillenkamp, Frank Puppe, Nils A. Loewen

**Affiliations:** 1 Department of Ophthalmology, University of Würzburg, Würzburg, Germany; 2 Institute for Artificial Intelligence and Knowledge Systems, Department of Informatics, University of Würzburg, Würzburg, Germany; 3 Artemis Eye Centers, Frankfurt, Germany

**Keywords:** glaucoma progression, nycthemeral intraocular pressure, mean ocular perfusion pressure

## Abstract

**Purpose:**

Nycthemeral (24-hour) intraocular pressure (IOP) monitoring in glaucoma has been used in Europe for more than 100 years to detect peaks missed during regular office hours. Data supporting this practice are lacking, because it is difficult to correlate manually drawn IOP curves to objective glaucoma progression. To address this, we developed an automated IOP data extraction tool, HIOP-Reader.

**Methods:**

Machine learning image analysis software extracted IOP data from hand-drawn, nycthemeral IOP curves of 225 retrospectively identified patients with glaucoma. The relationship between demographic parameters, IOP, and mean ocular perfusion pressure (MOPP) data to spectral-domain optical coherence tomography (SDOCT) data was analyzed. Sensitivities and specificities for the historical cutoff values of 15 mm Hg and 22 mm Hg in detecting glaucoma progression were calculated.

**Results:**

Machine data extraction was 119 times faster than manual data extraction. The IOP average was 15.2 ± 4.0 mm Hg, nycthemeral IOP variation was 6.9 ± 4.2 mm Hg, and MOPP was 59.1 ± 8.9 mm Hg. Peak IOP occurred at 10 am and trough at 9 pm. Progression occurred mainly in the temporal-superior and temporal-inferior SDOCT sectors. No correlation could be established between demographic, IOP, or MOPP variables and disease progression on OCT. The sensitivity and specificity of both cutoff points (15 and 22 mm Hg) were insufficient to be clinically useful. Outpatient IOPs were noninferior to nycthemeral IOPs.

**Conclusions:**

IOP data obtained during a single visit make for a poor diagnostic tool, no matter whether obtained using nycthemeral measurements or during outpatient hours.

**Translational Relevance:**

HIOP-Reader rapidly extracts manually recorded IOP data to allow critical analysis of existing databases.

## Introduction

The need for better diagnostic options in glaucoma is critical, as this disease only presents symptoms at an advanced stage and is often diagnosed late.^1^ Forty-two percent of all patients with primary open-angle glaucoma (POAG) ultimately go blind in one eye,^2^ partially because of this. To better assess the effectiveness of the treatment and to detect pressure peaks that are not recognized during office hours,^3^ patients in German-speaking countries are often admitted for nycthemeral (24-hour) intraocular pressure (IOP) profiles.^4^ Such monitoring generates costs averaging €643 per night^5^^,^^6^ and has been obtained, based on verbally communicated past use patterns at many clinics, at least approximately one million times in the past 100 years^4^^,^^7^^–^^9^ in the hopes of aiding the diagnosis and treatment of glaucoma. However, evidence supporting 24-hour IOP profiles for identifying IOPs above target or larger than normal IOP fluctuations^4^^,^^8^^–^^11^ is at most expert opinion (level V).^12^^–^^14^ The absence of strong evidence for 24-hour IOP profiles as a diagnostic tool in glaucoma is surprising, considering the contrast to the high-quality level I evidence that establishes IOP as the preeminent cause of glaucoma.^12^^–^^14^ Damage from high IOP is an experimentally demonstrated pathomechanism of glaucoma in nonhuman primates.^15^^,^^16^ Elevated IOP levels are strongly correlated to human glaucoma incidence,^17^^,^^18^ and their treatment reduces glaucoma onset and progression.^19^^,^^20^ Moreover, IOP fluctuations and pressure peaks during outpatient clinic hours have previously been associated with glaucoma progression.^21^

One reason for the missing link between vast historical records of 24-hour IOP profiles and glaucoma progression may be the difficulty in extracting data from manually drawn IOP curves that are paper based and correlating them to objective, statistically significant progression. To address this, we created a computer-aided image analysis of 24-hour IOP profiles. We matched them to worsening retinal nerve fiber layer thickness using current spectral-domain optical coherence tomography (SDOCT) and software (SPECTRALIS SDOCT; Heidelberg Engineering, Heidelberg, Germany). Similarly, we estimated the ocular perfusion pressure and determined the strength of correlation to progression.

High IOP damages the axons of retinal ganglion cells primarily at the level of the lamina cribrosa, a biomechanical weak point.^22^^,^^23^ Too low an ocular perfusion pressure^24^ is considered a secondary contributing factor.

Based on the well-honed, century-old practice of obtaining inpatient IOPs, our primary hypothesis was that 24-hour inpatient IOPs would be expected to be correlated to a statistically significant decline of the retinal nerve fiber layer (RNFL), in particular the temporal-superior, temporal or temporal-inferior RNFL. Our secondary hypothesis was that ocular perfusion pressure is correlated to glaucoma progression.

## Methods

### Study Design

This retrospective chart review was carried out at the Department of Ophthalmology of the University of Würzburg. It abided by the principles stated in the Declaration of Helsinki. Due to its retrospective nature, informed consent was waived by the Institutional Review Board of the University of Würzburg. Charts of 225 patients admitted to the ophthalmology inpatient unit at the University Hospital of Würzburg for nycthemeral IOP monitoring from 2017 to 2019 were analyzed to comprise 2 years since the introduction of OCT-aided progression analysis in this hospital. In 2019, the half-century-old practice of 24-hour measurements was halted and questioned under a new service director (NAL). Only right eyes were analyzed to reduce bias. Patients included had a diagnosis of POAG, low-tension glaucoma (LTG), pseudoexfoliation glaucoma (PXG), pigmentary glaucoma (PG), and juvenile open-angle glaucoma (JOAG). Patients with terminal, neovascular, uveitic, or angle-closure glaucoma were excluded from the study. Terminal glaucoma was defined as having a nearly complete visual field loss or a cup-to-disc ratio of 1.0.

**Figure 1.**
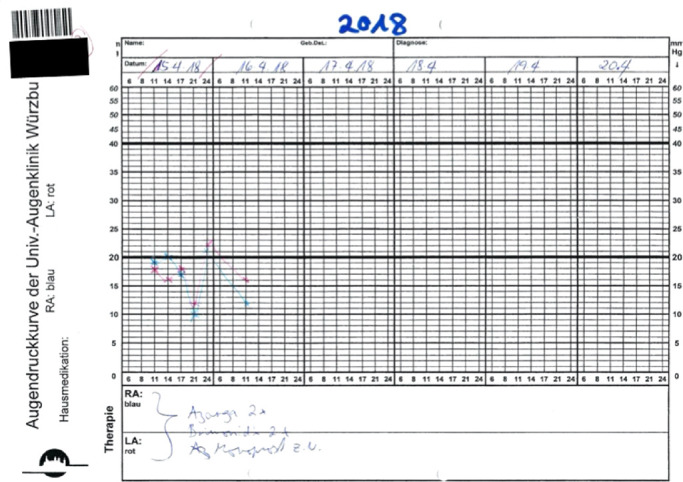
Example of an IOP chart used throughout the country of this study to this day. The time is displayed on a nonlinear x-axis with uneven intervals and the IOP on a nonlinear y-axis with a scale compressed above 40 mm Hg. The length of the x-axis of this chart template indicates that IOP curves were sometimes obtained for 6 days. *Blue* = right eye; *red* = left eye. A patient-identifying sticker is blacked out in the *left upper corner*.

Parameters recorded included age, gender, diagnosis, history of surgery, family history of glaucoma, medications, slit lamp, fundoscopic examination findings, and the central corneal thickness. The 24-hour IOP protocol established in this hospital called for measurements in the habitual position with 10 am, 2 pm, 5 pm, and 9 pm readings obtained by Goldmann applanation tonometry (Haag-Streit, Köniz, Switzerland) in the sitting position and the 12 am measurement obtained by Perkins applanation tonometry (Perkins MK3; Haag-Streit) in the supine position. IOPs were recorded on paper charts using blue for right eyes and red for left eyes, which is standard practice in Germany (Fig. 1). Each subject's 24-hour IOP data were fit to a cosine curve. Because there were only 5 measurements, instead of at least 12, this fit was done manually using a sparkline macro.^3^^,^^25^ The acrophase was estimated by defining it as the phase timing, in which a peak IOP during the 24 hours was reached. Paper-based 24-hour IOP profiles were examined using a custom-made computer-aided image analysis program. Values noted were T_max_, T_min_, T_avg_, and IOP_var_ (T_max_ – T_min_). Additionally, the mean ocular perfusion pressure (MOPP) was calculated as two-thirds of the difference between the mean arterial pressure and T_avg_.

### Image Analysis of Manually Recorded 24-Hour IOP Profiles

We wrote the Python-based program HIOP-Reader^26^ to extract patient name, examination date, and the IOP values on the y-axis with their corresponding time on the x-axis. We used OpenCV^27^ for image processing, Tesseract^28^ for optical character recognition, and TensorFlow^29^ and scikit-learn^30^ for machine learning. The image analysis was divided into three parts: preprocessing, value detection, and name and date extraction.

The main goal of preprocessing was to detect the frame containing the IOP profile and crop the image to it. We achieved this by searching for curves, joining all continuous points with the same intensities. In OpenCV, this is referred to as contours. To improve the accuracy of finding contours, we binarized the image by applying adaptive thresholding. We used Gaussian adaptive thresholding, which calculates the Gaussian weighted sum over a neighborhood of, in our case, 27 × 27 pixels, to find an appropriate threshold value. This threshold, minus a constant C = 10, was then used to binarize the image. From the binary image, we chose the largest resulting contour as the main frame of the image. To make the process more robust, we ensured that the resulting contour is a rectangle. This was done by approximating the contour using the Douglas–Peucker algorithm,^31^^,^^32^ ensuring that the contour consisted of four lines even when the frame was cut off or other artifacts were obstructing the frame. Next, we checked the angles between the four lines of the approximated contour, ensuring that it was at least close to a rectangle. Finally, we cropped the image to the resulting approximation of the largest contour, resulting in an image cropped to the main frame of the IOP profile. After cropping, all scanned images had the same format and size, enabling us to do precise pixel position-based operations.

To extract the IOP values entered into the profile, we detected the lines representing the different examination times using the Canny edge detection algorithm^33^ and Hough line transformation.^34^ Any falsely detected or horizontal lines were removed. This left us with the precise positions of the lines representing different times. For each line, a neighborhood around it was considered when searching for IOP values. We exploited the fact that all IOP values for the left eye were entered in red, while all values for the right eye were entered in blue and created color-specific masks. These masks only contained the part of the image that was blue or red, respectively. IOP values were collected using these masks and the immediate vicinity of each line. Lastly, since all images had the same format, the IOP value could be directly inferred from the pixel position of the detected entry.

To capture the date of the 24-hour IOP profile, we applied a traditional machine learning approach. First, we isolated the area where the date was recorded and separated the numbers and the delimiters using contours. The numbers were then predicted using a convolutional neural network trained on the Modified National Institute of Standards and Technology (MNIST) data set.^35^ As the patient names were mostly recorded using machine-written labels, optical character recognition with Tesseract^28^ could be used to extract all machine-written text on the form. We used regular expressions on the extracted text to find patient names. All information was manually confirmed and stored as CSV files. To allow for rapid editing and error correction, we developed a graphical user interface for the program.

### Statistical Analysis

#### Data Management

Confirmatory and exploratory data analysis was performed using JMP (JMP 15.2.1; SAS Institute, Inc., Cary, NC, USA). Means along with standard deviations were calculated for continuous variables, while percentages were computed for categorical variables. A Kolmogorov–Smirnov test was run to assess continuous variables for a normal distribution. Bivariate analysis was used to study the relationship between various IOP parameters. Independent sample *t*-tests were used to compare means of continuous variables, whereas a χ^2^ test compared those of categorical variables. Spearman's rank-order correlation coefficient (rather than a Pearson's correlation coefficient) was reported if data sets were not normally distributed. For all our analyses, a *P* value of 0.05 or less was considered statistically significant.

#### OCT and Disease Progression Analysis

Disease progression was assessed using a SDOCT (SPECTRALIS OCT; Heidelberg Engineering GmbH). The RNFL thickness (in micrometers) of all peripapillary sectors was recorded. Changes in RNFL thickness were evaluated using commercial software (HEYEX Version 2.4.1; Heidelberg Engineering GmbH), which provided both the rate of RNFL loss and a statistical comparison to a normal age-related RNFL loss rate. In this way, progression was calculated both as a continuous and as a dichotomous variable. Linear regression was utilized to assess the relationship between several continuous variables (such as IOP_var_) and the rate of RNFL loss, representing disease progression. A contingency analysis was carried out to determine the sensitivity and specificity of using 15 and 22 mm Hg as T_max_ cutoff points in detecting disease progression in any sector. These sensitivity and specificity measurements were then calculated with 10 am, 2 pm, and 5 pm values to compare these values to a hypothetical outpatient situation.

## Results

**Figure 2.**
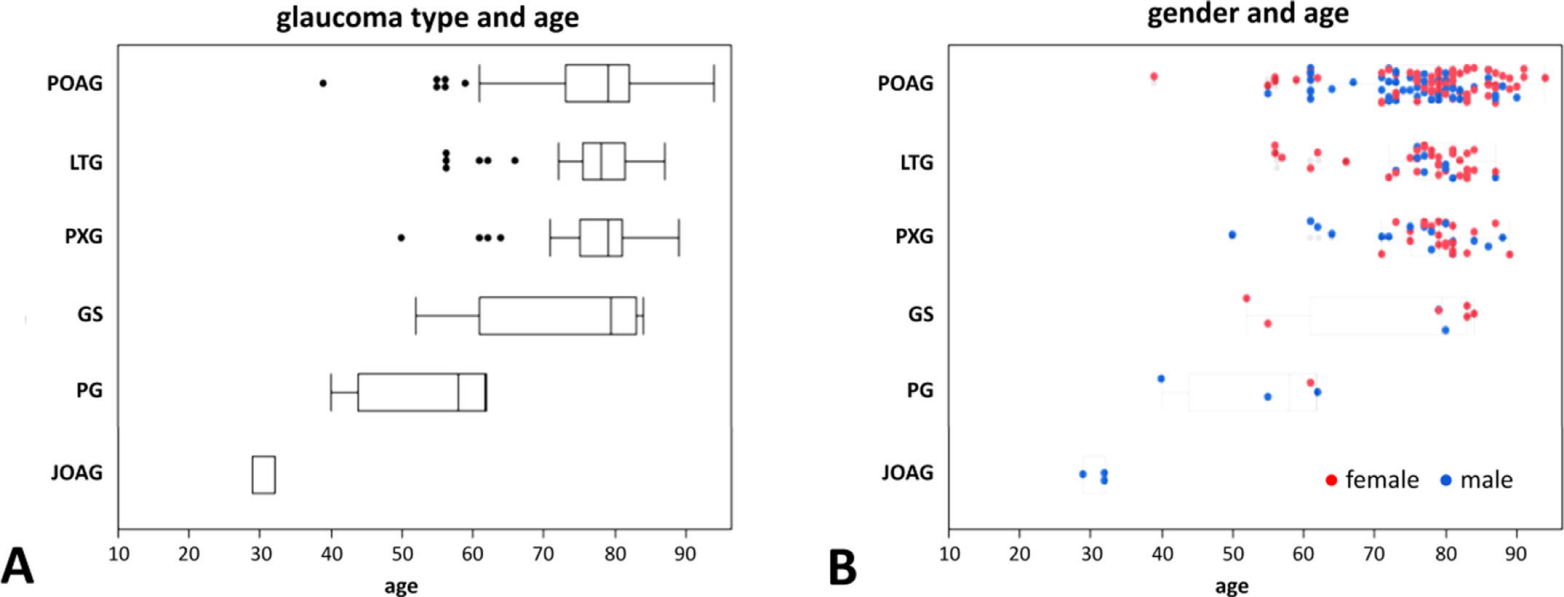
(**A**) Glaucoma type and age distribution. Patients with POAG, LTG, PXG, and GS had similar averages, while patients with PG were younger and those with JOAG were the youngest. (**B**) Gender and age distribution. There were disproportionately more female patients with LTG who were younger than male patients with LTG.

Table 1 depicts the demographic variables of the 225 patients included in this analysis. Five eyes were excluded due to meeting our criteria for terminal glaucoma. There were 137 women (61%) and 88 men (39%). Women were significantly older than men (77.0 ± 10.0 years vs. 72.8 ± 12.6 years, respectively, *P* = 0.006). The diagnoses included were POAG (*n* = 130, 57.8%), LTG (*n* = 41, 18.2%), PXG (*n* = 39, 17.3%), GS (Glaucoma suspects) (*n* = 8, 3.6%), PG (*n* = 4, 1.8%), and JOAG (*n* = 3, 1.3%). Patients with POAG, LTG, and PXG were older than those with PG and JOAG (*P* < 0.001; Fig. 2). Compared to the 3:2 ratio of women to men in this study, there were disproportionately more women (78%, *n* = 32) with LTG than men (22%, *n* = 9). There was no statistically significant difference in the number of medications per eye in both groups, with an average of 2.2 drops in each group (*P* = 1.0, Table 1). Fifty-eight patients had four different topical glaucoma medications, with prostaglandin analogues being the most prescribed medication (31.6%), followed by carbonic anhydrase inhibitors (27.0%), α-agonists (22.0%), and β-blockers (19.4%). The mean central corneal thickness (CCT) was 526.3 ± 35.7 µm. There was no gender difference (females: 538.6 ± 34.0 µm, males: 534.8 ± 38.3 µm, respectively, *P* = 0.43).

**Table 1.** Demographic Parameters of Included Patients

Characteristic	Males (*n* = 88)	Females (*n* = 137)	*P* Value	Total
Age (y)	72.8 ± 12.6	77.0 ± 10.0	0.006**^a^**	75.4 ± 11.2
Central corneal thickness (µm)	534.8 ± 38.3	538.6 ± 34.0	0.43	536.3 ± 35.7
Average number of drops	2.2 ± 1.6	2.2 ± 1.5	1.00	2.2 ± 1.5
Average number of surgeries	0.6 ± 0.7	0.6 ± 0.8	0.77	0.6 ± 0.7
T_avg_ (mm Hg)	15.9 ± 5.0	14.7 ± 3.1	0.03	15.2 ± 4.0
T_max_ (mm Hg)	20.3 ± 6.9	18.7 ± 4.0	0.03	19.3 ± 5.4
IOP_var_ (mm Hg)	7.4 ± 4.9	6.6 ± 3.7	0.17	6.9 ± 4.2
MOPP (mm Hg)	58.8 ± 9.0	59.3 ± 8.8	0.68	59.1 ± 8.9

a *P*-value < 0.05.

**Figure 3.**
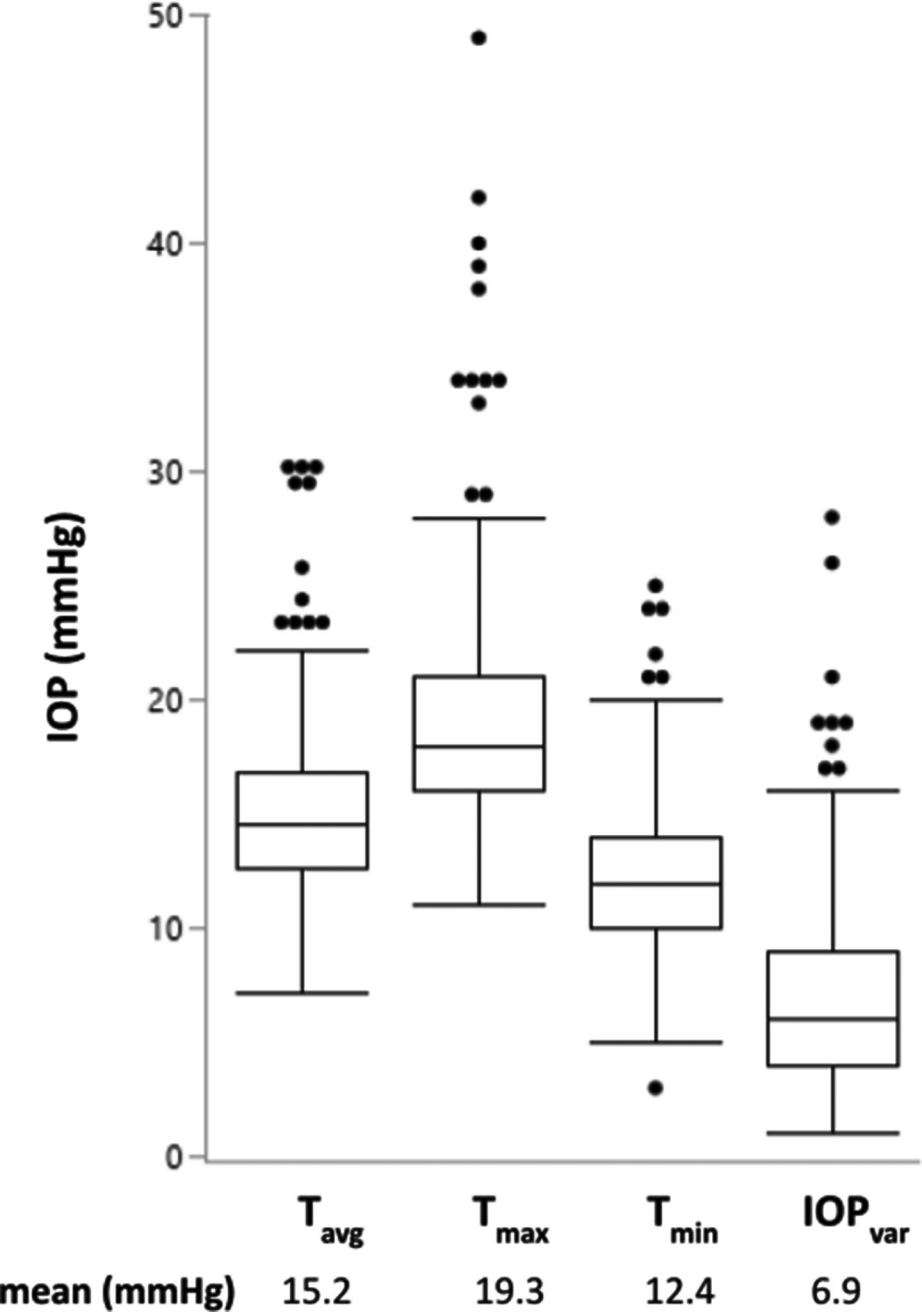
IOP average, maxima, minima, and variation.

We evaluated HIOP-Reader on 100 IOP profiles. An average of 3.60 ± 0.81 seconds was needed to process a file, not accounting for human error correction. In contrast, manual data extraction took 429.06 ± 96.61 seconds, or 119 times longer. The IOP curves showed a mean of 8.43 entries per eye. The names were detected correctly with an accuracy of 75.32%, and the detection of the date was only accurate in 42.85% of the cases. The entered values were detected with high accuracy. On average, there were 0.4675 falsely detected entries per IOP curve. Given the average of 8.43 entries per eye, this results in a false-positive rate of 5.54%. An average of 0.3376 entries per IOP curve were not detected, resulting in a false-negative rate of 4%. For the detected entries, the average distance between the actual value and the predicted value was 0.0927. We observed a mean value of 14.72 per entry, giving us a mean relative error of 0.63%. The evaluation was performed on standard consumer hardware from 2019 with a 2.4-GHz Quad-Core Intel Core i5-8279U CPU and 16 GB of random access memory. LTG had a significantly lower T_avg_ and T_max_ than POAG and PXG (*P* = 0.005 and *P* < 0.001, respectively; Fig. 3). The CCT of LTG was not significantly different from POAG or PXG (both *P* > 0.05). IOP_var_ was correlated with T_max_ (correlation 0.8, *P* < 0.001) and with T_avg_ (correlation 0.3, *P* < 0.001) but not with T_min_.

**Figure 4.**
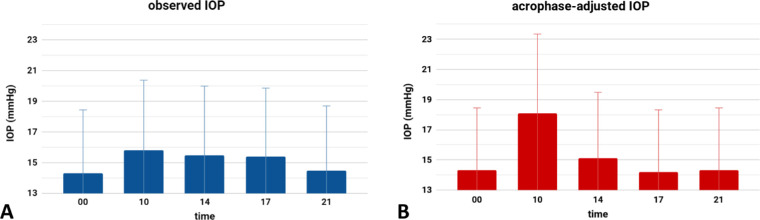
Nycthemeral (24-hour) IOPs as observed (A) and when arranged by estimated acrophases (B).

**Figure 5.**
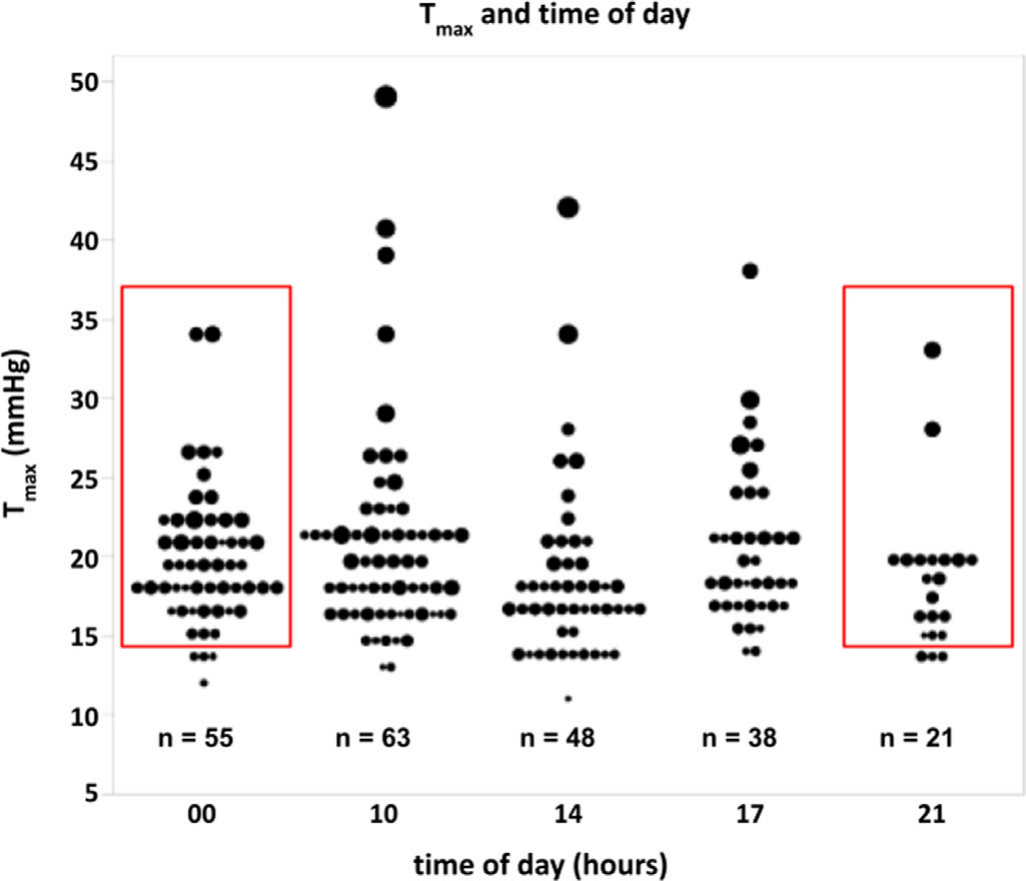
T_max_ and time of day at which T_max_ was reached. Each *bubble* represents the T_max_ of one patient during the 24-hour IOP inpatient measurement. The *bubble size* indicates the amount of 24-hour IOP variation. *Red boxes* indicate T_max_ measurements above 15 mm Hg that would not be detected during typical outpatient office hours.

The observed average IOPs were relatively similar throughout the day and ranged from a peak of 15.8 ± 5.1 mm Hg at 10 am to a trough of 14.5 ± 4.6 mm Hg at 9 pm (*P* = 0.519; Fig. 4). In total, 109 patients had an acrophase with peak IOP at 10 am. The acrophase spread was 8.4 ± 3.8 hours. When all 24-hour IOP curves were adjusted to have matching acrophases, a peak IOP of 18.1 ± 5.3 mm Hg was reached at 10 am and a trough of 14.2 ± 4.1 mm Hg at 9 pm (*P* < 0.001; Fig. 5).

**Figure 6.**
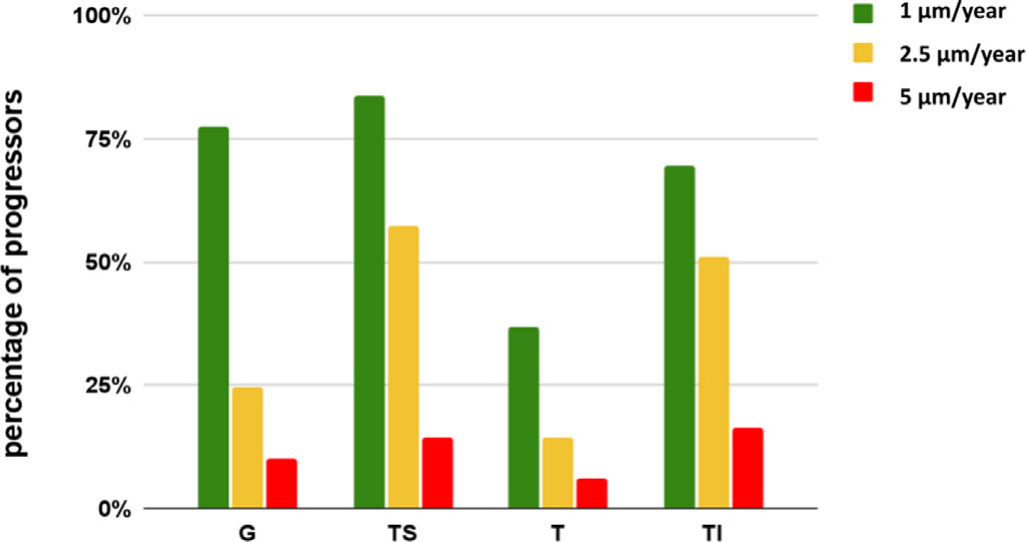
The percentage of progressors who had an RNFL loss of at least 1 (*green*), 2.5 (*yellow*), and 5 (*red*) µm per year. G, global peripapillary region; T, temporal quadrant; TI, temporal-inferior quadrant; TS, temporal-superior quadrant.

OCT progression data were available in 116 out of 225 patients. Of those, 42% were progressors with a significantly worsening retinal nerve fiber layer thickness. More progressors declined in the TI (31%) and TS (36%) sector than in T (22%; Fig. 6). Between progressors and nonprogressors, there were no differences in age, gender, or type of glaucoma, nor was there a difference in their IOP peak time, IOP_var_, T_max_, T_avg_, or T_min_ (all *P* > 0.05). IOP_var_ was 6.3 ± 3.6 mm Hg in progressors and 6.8 ± 3.9 mm Hg in nonprogressors, respectively. There was no difference in age. The RNFL decline in these progressors had an average of 2.3 ± 1.7 µm per year. Ten percent had a decline of more than 5 µm/y. Applying an old concept that IOP variations of more than 5 mm Hg may indicate glaucoma progression underlying the rationale of obtaining inpatient, 24-hour IOP measurements, sensitivity for such variation to detect glaucoma progression was 68% and specificity 25%.

**Figure 7.**
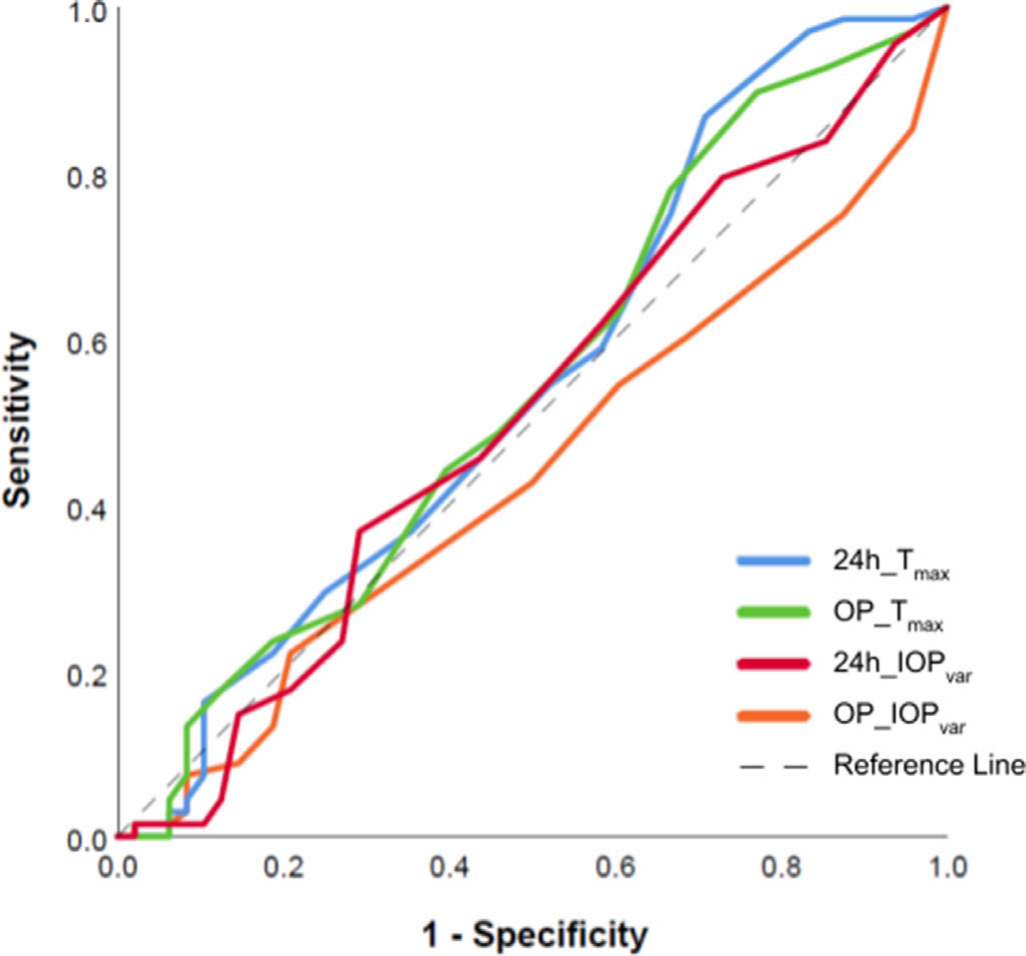
ROC curves comparing 24-hour and outpatient parameters of T_max_ and IOP_var_ for disease progression. IOP_var_ values of <5 mm Hg were excluded from the analysis. This figure shows a very low predictive power of disease progression for all parameters. Well-performing tests have a hyperbolic ROC curve with sensitivity and specificity close to 90%. 24h, nycthemeral measurements; OP, measurements during outpatient times.

Applying a historical cutoff of 22 mm Hg as an IOP considered too high, sensitivity was only 7%, and specificity was 87%. When a cutoff of 15 mm Hg was chosen, corresponding to a normal IOP of healthy eyes often viewed as suboptimal for moderate to advanced glaucoma, sensitivity was 69%, and specificity was 23%. Table 2 shows the sensitivity and specificity of those cutoff values obtained during 24-hour measurements and compares them to the same IOP criteria if those were applied to regular outpatient clinic hours. The specificity of the criteria “15 mm Hg” during outpatient hours was slightly better than when applied to inpatient 24-hour measurements, while the criteria “22 mm Hg” were very similar. Figure 7 applies the concept of T_max_ and T_avg_ as a test for glaucoma progression to a receiver operating characteristic (ROC) curve. All curves, regardless of inpatient or outpatient values, were close to the reference line, indicating poor performance.

**Table 2.** Comparison of Sensitivity and Specificity between Progression as Nominal Variable and T_max_ Measurements Using 15 and 22 mm Hg as Cutoff Values

Cutoff Value	Parameter	24-hour IOP	OP-IOP	Difference
15 mm Hg	Sensitivity	0.69	0.63	0.06
	Specificity	0.23	0.40	−0.17
22 mm Hg	Sensitivity	0.07	0.06	0.01
	Specificity	0.87	0.89	−0.02

OP-IOP, IOP measurements during outpatient hours (10 am, 2 pm, 5 pm).

Table 3 summarizes the correlations we found. T_max_, T_avg_, T_min_, and IOP_var_ were not correlated to the slope (speed) of RNFL loss (*P* > 0.05). These parameters were also not correlated to structural differences between the expected, normative RNFL thickness or the actual (absolute) RNFL thickness measured by the SPECTRALIS OCT.

**Table 3.** Correlation Coefficients for IOP and Progression

Characteristic	T_avg_	T_max_	T_min_	IOP_var_	MOPP
T_avg_	—				
T_max_	**0.74** **^a^**	—			
T_min_	**0.87** **^a^**	**0.54** **^a^**	—		
IOP_var_	0.11	**0.64** **^a^**	−**0.21****^a^**	—	
MOPP	−**0.14****^a^**	−**0.15****^a^**	−**0.14****^a^**	−0.025	—
G SL	−0.09	−0.04	0.06	−0.01	−0.05
TS SL	−0.04	−0.1	−0.15	<−0.01	−**0.09****^a^**
T SL	−0.05	−0.05	0.03	−0.04	−0.04
TI SL	−0.11	−0.09	−0.01	−0.01	−0.02

Spectralis OCT parameters: G SL, slope of global RNFL loss; TI SL, slope of temporal-inferior RNFL loss; T SL, slope of temporal RNFL loss; TS SL, slope of temporal-superior RNFL loss.

a significance *p*-value < 0.05.

The estimated MOPP was 59.1 ± 8.9 mm Hg. This parameter did not differ by glaucoma type (*P* = 0.42) or sex (*P* = 0.79). MOPP correlated negatively and weakly to the slope of the temporal-superior retinal fiber layer thickness (*r* = −0.09, *P* = 0.04), T_avg_ (*r* = −0.14, *P* = 0.04), T_max_ (*r* = −0.15, *P* = 0.03), and T_min_ (*r* = −0.14,*P* = 0.04) but not to IOP_var_ (*P* = 0.72). There was no significant correlation between MOPP and worsening glaucoma (*P* = 0.34). This was also not the case in LTG (*P* = 0.14).

## Discussion

We developed a high-efficiency reader specifically to extract nycthemeral IOP data from manually drawn charts and assessed disease progression using an SDOCT with progression analysis software. We found no significant relationship between nycthemeral IOP measurements and glaucoma progression, despite the best efforts.

HIOP-Reader allowed us to rapidly process and extract a large amount of image data with a low error rate. This program is made available to the scientific community via GitHub,^26^^,^^36^ a public software repository. Further improvements could be made with date extraction using component labeling and support vector machine classification^37^ or hidden Markov model^38^ based methods. The functionality that allows for statistical analysis of handwritten IOP profiles worked well. In particular, the program showed resilience to imperfections inherent to IOP profiles drawn manually by different users, and the IOP values were detected with high accuracy. This allowed us to process and use large amounts of handwritten data that would have been hard to acquire. We believe HIOP-Reader will be a useful mining tool to process the many decades of data available at inpatient-based ophthalmology clinics that have performed nycthemeral IOP measurements in the past.

Regarding patient demographics in our study, the gender ratio of women (61%) to men (39%) was very similar, almost down to the digit to that of global glaucoma studies.^39^^,^^40^ Among glaucoma subtypes, LTG, in particular, is more prevalent in women,^41^ a pattern seen in our study as well. Except for age, the demographic variables of men and women did not differ.

The idea behind collecting 24-hour IOPs appears to have been that patients with glaucoma might have a higher nocturnal peak and a larger IOP variation than normal eyes^4^^,^^7^^–^^9^^,^^11^ when, in fact, it has been known for a while that healthy eyes have a larger IOP variation than glaucomatous eyes.^42^ Looking for nocturnal peaks may also be of limited diagnostic value, as an elevated nocturnal IOP in the supine position is a physiologic reaction in healthy and glaucomatous eyes.^42^ Research into the relationship between IOP variation and glaucoma progression has produced discordant findings, however.^43^^–^^47^ A study of 105 POAG eyes with normal in-office IOP values showed IOP ranges over 5 days to be an independent risk factor for disease progression (defined as visual field loss).^43^ Similarly, some studies showed short-term (48-hour) and long-term IOP fluctuations to be correlated to visual field progression.^44^^,^^47^^,^^48^ Likewise, studies by Yang et al.^49^ and De Moraes et al.^50^ correlated 24-hour IOP measurements with a contact lens sensor to visual field deterioration in patients with POAG. This may indicate a superiority of continuous electronic IOP measurements in predicting glaucoma progression rather than manually collecting measurements at specific time intervals. Other investigators failed to corroborate intraocular pressure fluctuations and glaucoma progression.^45^^,^^46^ One reason for this may be the inclusion of patients with glaucoma undergoing medical therapy, who have a smaller fluctuation range.^51^ A 2007 study on 71 treated POAG eyes compared office IOP (9 am–6 pm) to 24-hour IOP readings and showed no statistical significance in the mean IOPs of both groups.^52^ In another study, the office IOP fluctuation was substantially lower than that of 24-hour measurements, and the two were not be correlated.^52^ Downs et al.^53^ found single measurements in nonhuman primates were not representative of complete profiles, and 24-hour profiles on one day were not reproducible on another day. Interestingly, a different study found that the mean outpatient IOP could, in fact, be used to predict both mean and peak nycthemeral IOPs.^54^

We found nycthemeral and office IOP variables to have an inadequate sensitivity and specificity in identifying progressors, as the ROC curves demonstrate. Well-performing medical diagnostic tests, such as the SDOCT, have a value close to 90% in both parameters (resulting in a hyperbolic curve shape).^55^ This does not mean that there is no connection between 24-hour IOP variables and glaucoma progression. Instead, our findings highlight the challenges of implementing a well-intended test in a busy clinical environment without the proper methods. The retrospective IOP data we processed in this study had considerable shortcomings. Values were recorded with a commitment to seemingly arbitrarily set times, unevenly distributed throughout the day, and at an interval larger than the 2-hour interval of IOP sleep lab studies.^3^^,^^25^ Such a customized schedule might fit clinicians’ work schedules better, but it prevents finding the best-fitting cosine curve and the peak (acrophase) as the phase timing of the 24-hour rhythm.^25^ The IOP peak at 10 am in our data appeared to be later than in previous studies, but this is unlikely to be the actual phase timing. Other studies reported peaks around 5:30 am,^56^ 6 am,^57^ and 8 am,^58^ and troughs at 2 pm,^58^ 5 pm,^44^ and 9:30 pm,^56^ respectively.

We found MOPP to be negatively correlated to T_avg_, T_max_, and T_min_. This is not surprising, as one would expect the perfusion pressure to increase somewhat as the IOP decreases. Our MOPP did not correlate to progression, on the other hand, as suggested by other studies that examined POAG, PXG, and LTG.^19^^,^^59^^–^^61^ A reduced nocturnal ocular perfusion pressure, in particular, has been associated with increased structural damage and visual field deterioration in patients with LTG.^59^^,^^62^ The blood pressure readings we used for the MOPP estimation were obtained on admission during late morning hours, however.

Our study points to several problems with the practice of obtaining 24-hour inpatient IOPs that concern rationale, data acquisition, and validity. Consequently, these issues became limitations of our study. We question the rationale of subjecting a patient to nycthemeral IOP measurements when it is often already known that there is an objective decline on SDOCT. It is difficult to see how a 24-hour IOP profile could be used to argue against advancing therapy.

Nocturnal data in the supine position were acquired using a Perkins tonometer. Although it can be as accurate as Goldmann applanation tonometry,^63^ it is highly operator dependent and requires experience that not all on-call residents might have. A pneumatonometer,^64^ a well-accepted standard for 24-hour IOP studies with high accuracy and reproducibility, would have been a better choice.^42^^,^^65^ Additionally, IOP was measured at irregular intervals (10 am, 2 pm, 5 pm, 9 pm, and 12 am) with no measurements taken during the middle of the sleeping period. This results in an incomplete characterization of the nycthemeral IOP profile that is scarcely more useful than single IOP measurements taken during clinic hours.

It is questionable whether the values measured during an inpatient stay are valid and reflect values at home because a clinic environment with close observation likely improves drop compliance. Diurnal intraocular pressure patterns have also been shown to be neither sustained nor reproducible.^66^ Since the role of IOP fluctuation in glaucoma progression is not well understood, it is unclear what characteristics of a single nycthemeral IOP profile could be considered equally to progressive structural damage on SDOCT in clinical relevance. This study might have come to a different conclusion if more patients had been available than the 225 included in this analysis. But if a clinical test does not already show a strong relationship between variables measured and disease in one individual, it is of little clinical use.

Given these issues, it is surprising that the practice of obtaining nycthemeral IOP profiles has been continued for more than a century. Answers might perhaps be found in how this practice appears to be limited to countries that could follow the literature on that topic in German^4^^,^^7^^–^^10^^,^^67^ and how these continue to favor inpatient reimbursements,^68^ although ophthalmology started to become an outpatient specialty in the late 1980s.^69^^–^^72^

In conclusion, we created software that acquired nycthemeral IOP data from hand-drawn IOP charts and performed at more than 100 times the speed of manual extraction. This study generated new pilot data on inpatient IOPs and related SDOCT variables. No correlation could be found between any IOP parameters or MOPP and objective glaucoma progression. ROC curves indicated a poor performance of 24-hour inpatient IOPs as a diagnostic tool.
